# Supplementary material for: Meeting the 2030 END TB goals in the wake of COVID-19: A modelling study of countries in the USAID TB portfolio
Source: PLOS Glob Public Health. 2023 Oct 23;3(10):e0001271. doi: 10.1371/journal.pgph.0001271 (PMC10593207; doi:10.1371/journal.pgph.0001271)
Supplement: S1 Text — (DOCX) [file pgph.0001271.s001.docx]

**Meeting the 2030 End TB goals in the wake of COVID-19: a modelling study of countries in the USAID TB portfolio**

**Supporting information**

Table of Contents

[1. Governing equations 1](#_Toc116911608)

[1.1 South Asia model 1](#_Toc116911609)

[1.2 Sub-Saharan Africa model 5](#_Toc116911610)

[1.3 Central Asia/Europe model 10](#_Toc116911611)

[2. Model calibration 14](#_Toc116911612)

[3. Intervention descriptions 15](#_Toc116911613)

[4. Supplementary figures and tables 17](#_Toc116911614)

[5. Implications of the 2018 UN HLM targets for case detection 20](#_Toc116911615)

[6. References 23](#_Toc116911616)

# 1. Governing equations

While several modelling studies have addressed intervention priorities to meet the SDG goals for TB [1,2], our current analysis does so while also incorporating the adverse effects of disruptions to TB services during the COVID-19 pandemic. We follow a similar approach as recently employed in the Global Plan to End TB, 2023 – 2030 [3], but with a focus on the countries in the USAID portfolio. As described in the main text, we aggregated these countries into three country groups: South Asia, Sub-Saharan Africa, and Central Asia/Europe. In the following list of governing equations for each country group, the accompanying tables ( A - C) provide definitions and estimates of each of the model parameters.

## 1.1 South Asia model

Uninfected (U):

$$\frac{dU}{dt}=b-(\lambda+\mu)U$$

Latent, ‘fast’ progression ($L_{f}$):

$$\frac{dL_{f}}{dt}=\lambda U+c\lambda(L_{s}+R_{lo}+R_{hi}+R)-\left( u+v+\mu\right)L_{f}$$

Latent, ‘slow’ progression ($L_{s}):$

$$\frac{dL_{s}}{dt}=vL_{f}-\left( w+\mu+c\lambda\right)L_{s}$$

Active TB ($I$):

$$\frac{dI}{dt}=uL_{f}+wL_{s}+\rho_{lo}R_{lo}+\rho_{hi}R_{hi}+\rho_{st}R_{st}-\left( \mu_{TB}+\sigma+\gamma\right)I$$

Presented for care and awaiting diagnosis with public or private sector ($D^{\left( s \right)}$, with $s$ being $pu$ and $pr$ to denote the public and private sectors, respectively):

$$\frac{dD^{\left( s \right)}}{dt}=p_{sec}^{\left( s \right)}\left( \gamma I+\tilde{\gamma}E \right)-\left( \sigma+\mu_{TB}+md \right)D^{(s)}$$

Temporarily dropped out of careseeking due to missed diagnosis ($E$):

$$\frac{dE}{dt}=\sum_{s} md\left( 1-p_{Dx}^{\left( s \right)} \right)D^{\left( s \right)}-\left( \sigma+\mu_{TB}+\tilde{\gamma} \right)E$$

Diagnosed and initiated on treatment in sector *s* ($T^{(s)}$):

$$\frac{dT^{\left( s \right)}}{dt}=mdp_{Dx}^{\left( s \right)}D^{\left( s \right)}-\left( \tau+\delta^{\left( s \right)} \right)T^{\left( s \right)}$$

Recovered after treatment completion, with low relapse risk ($R^{\left( lo \right)}$):

$$\frac{dR_{lo}}{dt}=\tau\left[ T^{\left( pu \right)}+T^{\left( pr \right)} \right]-\left( \mu+\rho_{lo}+c\lambda+h \right)R_{lo}$$

Recovered after treatment non-completion with high relapse risk ($R_{hi}$):

$$\frac{dR_{hi}}{dt}=\delta^{(pu)}T^{\left( pu \right)}+\delta^{\left( pr \right)}T^{\left( pr \right)}-\left( \mu+\rho_{hi}+c\lambda+h \right)R_{hi}$$

Long-term recovered, following stabilisation of relapse risk ($R_{st}$):

$$\frac{dR_{st}}{dt}=h\left[ R_{lo}+R_{hi} \right]-\left( \mu+\rho_{st}+c\lambda\right)R_{st}$$

Force-of-infection ($\lambda):$

$$\lambda=\beta(I+D^{\left( 1 \right)}+D^{\left( 2 \right)}+E)$$

**Table A. Table of parameters for the South Asia model**

| Symbol | Meaning | | Value | Source |
| --- | --- | --- | --- | --- |
| Natural history | | | | |
| $\boldsymbol{\beta}$ | Transmission rate (annual number of infections per case) | | 24.0  [18.3 – 28.8] | Model calibration |
| $\boldsymbol{u}$ | Per-capita rate of ‘fast’ progression to active TB | | 0.081  [0.073 – 0.098] | Menzies et al (2018) ([4]), assuming a uniform prior distribution over +/- 10% of reported values |
| $\boldsymbol{v}$ | Per-capita rate of stabilisation from ‘fast’ to ‘slow’ latent states | | 0.79  [0.66 – 0.94] |  |
| $\boldsymbol{w}$ | Per-capita rate of ‘fast’ progression to active TB | | 0.00045  [0.00044 – 0.00046] |  |
| $\boldsymbol{\mu}_{\boldsymbol{TB}}$ | Per-capita rate of death from untreated TB | | 0.14  [0.08 – 0.35] | Tiemersma et al (2011) [5], with assumed uniform priors over a range of +/-25% |
| $\boldsymbol{\sigma}$ | Per-capita rate of self-cure from untreated TB | | 0.15  [0.18 – 0.21] |  |
| $\boldsymbol{\rho}_{\boldsymbol{lo}}$ | Per-capita rate of relapse in two years following treatment completion | | 0.034  [0.029 – 0.039] | Romanowski (2019)[6], Menzies (2009) [7] and Weis (1994) [8], with uniform prior using intervals of ± 50% |
| $\boldsymbol{\rho}_{\boldsymbol{hi}}$ | Per-capita rate of relapse in two years following treatment non-completion, or self-cure | | 0.12  [0.11 – 0.16] |  |
| $\boldsymbol{\rho}_{\boldsymbol{st}}$ | Per-capita rate of relapse beyond two years post treatment/self-cure | | 0.0014  [0.0011 – 0.0018] | Most relapse occurs in first two years after recovery: Guerra-Assuncao (2015) [9] |
| $\boldsymbol{h}$ | Per-capita annual rate of ‘stabilising’ from high to low relapse risk | | 0.5 |  |
| $\boldsymbol{c}$ | Reduced risk of reinfection arising from prior exposure | | 0.44  [0.28 – 0.66] | Andrews et al (2012) [10], with assumed uniform prior over range of [0.5 – 0.9] |
| Health services | | | | |
| $\boldsymbol{\gamma}$ | Per-capita rate of first careseeking, active TB | | 1.45  [0.74 – 3.04] | Model calibration, assuming uniform prior range of [0.1 – 100] |
| $\tilde{\boldsymbol{\gamma}}$ | Per-capita rate of repeat careseeking, active TB | | 10.9  [1.32 – 17.2] | Model calibration, assuming uniform prior range of [1 – 24] |
| $\boldsymbol{p}_{\boldsymbol{sec}}^{\left( \boldsymbol{s} \right)}$ | Per careseeking attempt, probability of choosing provider type *s* | Public sector ($s=0$)  Private sector ($s=1$) | 0.66  [0.55 – 0.70] $p_{sec}^{\left( 1 \right)}=1-p_{sec}^{\left( 0 \right)}$ by definition | Model calibration, assuming prior uniform range of [0 – 1] |
| $\boldsymbol{m}$ |  | |  |  |
| $\boldsymbol{d}$ | Per-capita rate of provider offering diagnosis | | 52 | Assumption, equivalent to 1 week |
| $\boldsymbol{p}_{\boldsymbol{Dx}}^{\left( \boldsymbol{s} \right)}$ | Per patient visit to a provider, probability of being correctly diagnosed with TB | | Public sector ($s=0)$: 0.81 [0.75 – 0.89] | Assumed uniform prior of [0.75, 0.90] |
|  |  |  | Private sector ($s=1)$: 0.45 [0.42 – 0.52] | Assumed uniform prior of [0.40, 0.70] |
| $\boldsymbol{\tau}$ | Per-capita rate of first-line treatment completion | | 2 | Corresponds to treatment duration of 6 months |
| $\boldsymbol{\delta}^{\left( \boldsymbol{s} \right)}$ | Per-capita rate of first-line treatment interruption | | Public sector ($s=0$): xx | Assumed treatment completion follows uniform prior of 0.75 – 0.95 |
|  |  |  | Private sector ($s=1$): xx | Assumed treatment completion follows uniform prior of 0.40 – 0.80 |
| Demographics | | | | |
| $\boldsymbol{\mu}$ | Per-capita rate of background (non-TB-related) mortality | | 1/70 | Mean lifespan of 70 years (World Bank) |
| $\boldsymbol{b}$ | Rate of population replenishment through birth | | Chosen to maintain constant population by balancing all deaths | |

## 1.2 Sub-Saharan Africa model

In the following equations, the superscript $j$ denotes HIV status, with values 0, 1, 2 denoting respectively: HIV-negative, HIV-positive but untreated, and on ART. The terms $f\left( . \right)$ represent transitions between different HIV strata, as described below.

Uninfected (U):

$$\frac{dU^{\left( j \right)}}{dt}=b\iota^{\left( j \right)}-(\lambda+\mu^{\left( j \right)})U^{\left( j \right)}+f(U^{\left( j \right)})$$

where $\iota^{\left( j \right)}$ is an indicator function, taking value 1 when $j=0,$ and 1 otherwise.

Latent, ‘fast’ progression ($L_{f}$):

$$\frac{dL_{f}^{\left( j \right)}}{dt}=\lambda U^{(j)}+c^{\left( j \right)}\lambda\left( L_{s}^{\left( j \right)}+R_{lo}^{\left( j \right)}+R_{hi}^{\left( j \right)}+R^{\left( j \right)} \right)-\left( u+v^{\left( j \right)}+\mu^{\left( j \right)} \right)L_{f}^{\left( j \right)}+f(L^{\left( j \right)})$$

Latent, ‘slow’ progression ($L_{s}):$

$$\frac{dL_{s}^{\left( j \right)}}{dt}=v{L^{\left( j \right)}}_{f}-\left( w+\mu^{\left( j \right)}+c\lambda\right)L_{s}^{\left( j \right)}+f(L_{s}^{\left( j \right)})$$

Active TB ($I$):

$$\frac{dI^{\left( j \right)}}{dt}=uL_{f}^{\left( j \right)}+wL_{s}^{\left( j \right)}+\rho_{lo}R_{lo}^{\left( j \right)}+\rho_{hi}R_{hi}^{\left( j \right)}+\rho_{st}R_{st}^{\left( j \right)}-\left( \mu_{TB}^{\left( j \right)}+\sigma^{\left( j \right)}+\gamma\right)I^{\left( j \right)}+f(I^{\left( j \right)})$$

Presented for care and awaiting diagnosis:

$$\frac{dD^{\left( j \right)}}{dt}=\gamma I^{\left( j \right)}+\tilde{\gamma}E^{\left( j \right)}-\left( \sigma^{\left( j \right)}+\mu_{TB}^{\left( j \right)}+md \right)D^{\left( j \right)}+f(D^{\left( j \right)})$$

Temporarily dropped out of careseeking due to missed diagnosis ($E$):

$$\frac{dE^{\left( j \right)}}{dt}=md\left( 1-p_{Dx} \right)D^{\left( j \right)}-\left( \sigma^{\left( j \right)}+\mu_{TB}^{\left( j \right)}+\tilde{\gamma} \right)E^{\left( j \right)}+f(E^{\left( j \right)})$$

Diagnosed and initiated on TB treatment ($T^{\left( j \right)}$):

$$\frac{dT^{\left( j \right)}}{dt}=mdp_{Dx}D^{\left( j \right)}-\left( \tau+\delta+\mu^{\left( j \right)} \right)T^{\left( j \right)}+f(T^{\left( j \right)})$$

Recovered after treatment completion, with low relapse risk ($R^{\left( j, lo \right)}$):

$$\frac{dR_{lo}^{\left( j \right)}}{dt}=\tau T^{\left( j \right)}-\left( \mu^{\left( j \right)}+\rho_{lo}^{\left( j \right)}+c^{\left( j \right)}\lambda+h \right)R_{lo}^{\left( j \right)}+f(R_{lo}^{(j)})$$

Recovered after treatment non-completion with high relapse risk ($R_{hi}^{\left( j \right)}$):

$$\frac{dR_{hi}^{\left( j \right)}}{dt}=\delta T^{\left( j \right)}-\left( \mu^{\left( j \right)}+\rho_{hi}^{\left( j \right)}+c^{\left( j \right)}\lambda+h \right)R_{hi}^{\left( j \right)}+f(R_{hi}^{(j)})$$

Long-term recovered, following stabilisation of relapse risk ($R^{\left( j,st \right)}$):

$$\frac{dR_{st}^{\left( j \right)}}{dt}=h\left[ R_{lo}^{\left( j \right)}+R_{hi}^{\left( j \right)} \right]-\left( \mu^{\left( j \right)}+\rho_{st}^{\left( j \right)}+c^{\left( j \right)}\lambda\right)R_{st}^{\left( j \right)}+f(R_{st}^{(j)})$$

Force-of-infection $(\lambda)$:

$$\lambda=\beta\left[ \left( I^{\left( 0 \right)}+D^{\left( 0 \right)}+E^{\left( 0 \right)} \right)+q_{hiv}\sum_{j\in[1,2]} \left( I^{\left( j \right)}+D^{\left( j \right)}+E^{\left( j \right)} \right) \right]$$

For the terms $f(.)$ representing transitions between different HIV strata, we have, for a given state variable $X^{\left( j \right)}$:

$$f\left( X^{\left( j \right)} \right)=\left\{ \begin{matrix} -r_{HIV}X^{\left( 0 \right)} & j=0 \\ r_{HIV}X^{\left( 0 \right)}-r_{ART}X^{\left( 1 \right)} & j=1 \\ r_{ART}X^{\left( 1 \right)} & j=2 \end{matrix} \right.$$

where $r_{HIV}, r_{ART}$ are, respectively, time-dependent rates of HIV acquisition, and of ART initiation.

**Table B. Table of parameters for the Sub-Saharan Africa model**

| Symbol | Meaning | | | Value | Source |
| --- | --- | --- | --- | --- | --- |
| Natural history | | | | | |
| $\boldsymbol{\beta}$ | Transmission rate (annual number of infections per case), HIV -ve TB | | | 21.8  [8.59 – 29.4] | Model calibration |
| $\boldsymbol{q}_{\boldsymbol{HIV}}$ | Relative transmission rate, HIV+ve TB relative to HIV-ve | | | 0.37  [0.01 – 0.93] |  |
| $\boldsymbol{u}_{\boldsymbol{h}}$ | Per-capita rate of ‘fast’ progression to active TB | $j=0$ (HIV -ve) | | 0.081  [0.073 – 0.098] | Menzies et al (2018) ([4]), assuming a uniform prior distribution over +/- 10% of reported values |
|  |  | $j=1$ (HIV +ve, not on ART) | | $u_{1}=k_{LTBI}u_{0}$, for $k_{LTBI}$ to be calibrated (see below) | |
|  |  | $j=2$ (on ART) | | $u_{2}=u_{1}\left( 0.4-0.24p \right),$ where $p$ is the coverage of IPT amongst those on ART, and assuming that ART and IPT independently have 60% effectiveness in reducing TB incidence. Formula derived as a weighted average (weighted by $p)$ of progression rates depending on TPT status | |
| $\boldsymbol{v}_{\boldsymbol{h}}$ | Per-capita rate of stabilisation from ‘fast’ to ‘slow’ latent states | $j=0$ (HIV -ve), $j=2$   (on ART) | | 0.79  [0.66 – 0.94] | Menzies et al (2018) ([4]), assuming a uniform prior distribution over +/- 10% of reported values |
|  |  | $j=1$ (HIV+ve, not on ART) | | 0 | Assumption: without ART, PLHIV have no ‘stabilisation’ of progression risk |
| $\boldsymbol{w}_{\boldsymbol{h}}$ | Per-capita rate of ‘slow’ progression to active TB | $j=0$ (HIV -ve) | | 0.00045  [0.00044 – 0.00046] | Menzies et al (2018) ([4]), assuming a uniform prior distribution over +/- 10% of reported values |
|  |  | $j=1$ (HIV +ve, not on ART) | | $u_{1}=k_{LTBI}u_{0}$, for $k_{LTBI}$ to be calibrated (see below) | |
|  |  | $j=2$ (on ART) | | $w_{2}=w_{1}(0.4-0.24p)$, where $p$ is the coverage of IPT amongst those on ART, and assuming that ART and IPT independently have 60% effectiveness in reducing TB incidence. Formula derived as a weighted average (weighted by $p)$ of progression rates depending on TPT status. | |
| $\boldsymbol{k}_{\boldsymbol{LTBI}}$ | Relative rate of progression to active TB, HIV+ve (untreated) vs HIV -ve | | | 45.2  [6.65 – 90.8] | Calibrated, to match HIV/TB incidence |
| $\boldsymbol{\mu}_{\boldsymbol{TB}}^{\left( \boldsymbol{j} \right)}$ | Per-capita rate of death from untreated TB | $j=0$ (HIV -ve) $j=2$   (on ART) | | 0.28  [0.13 – 0.33] | Tiemersma et al (2011) [5], with assumed uniform priors over a range of +/-25% |
|  |  | $j=1$ (HIV +ve, not on ART) | | 0.52  [0.16 – 1.16] | Calibrated to match HIV/TB mortality estimates |
| $\boldsymbol{\sigma}^{\left( \boldsymbol{j} \right)}$ | Per-capita rate of self-cure from untreated TB | $h=0$ (HIV -ve) $h=2$   (on ART) | | 0.18  [0.14 – 0.19] | Tiemersma et al (2011) [5], with assumed uniform priors over a range of +/-25% |
|  |  | $h=1$ (HIV +ve, not on ART) | | 0 | Assumption |
| $\boldsymbol{\rho}_{\boldsymbol{lo}}$ | Per-capita rate of relapse in two years following treatment completion | | | 0.034  [0.029 – 0.039] | Romanowski (2019)[6], Menzies (2009) [7] and Weis (1994) [8], with uniform prior using intervals of ± 50% |
| $\boldsymbol{\rho}_{\boldsymbol{hi}}$ | Per-capita rate of relapse in two years following treatment non-completion, or self-cure | | | 0.12  [0.11 – 0.16] |  |
| $\boldsymbol{\rho}_{\boldsymbol{st}}$ | Per-capita rate of relapse beyond two years post treatment/self-cure | | | 0.0014  [0.0011 – 0.0018] | Most relapse occurs in first two years after recovery: Guerra-Assuncao (2015) [9] |
| $\boldsymbol{h}$ | Per-capita annual rate of ‘stabilising’ from high to low relapse risk | | | 0.5 |  |
| $\boldsymbol{c}$ | Reduced risk of reinfection arising from prior exposure | | | 0.64  [0.54 – 0.86] | Andrews et al (2012) [10], with assumed uniform prior over range of [0.5 – 0.9] |
| Health services | | | | | |
| $\boldsymbol{\gamma}$ | Per-capita rate of first careseeking, active TB | | | 1.15  [0.33 – 1.76] | Model calibration, assuming uniform prior range of [0.1 – 100] |
| $\tilde{\boldsymbol{\gamma}}$ | Per-capita rate of repeat careseeking, active TB | | | 10.1  [1.75 – 21.7] | Model calibration, assuming uniform prior range of [1 – 24] |
| $\boldsymbol{m}$ |  | | |  |  |
| $\boldsymbol{d}$ | Per-capita rate of provider offering diagnosis | | | 52 | Assumption, equivalent to 1 week |
| $\boldsymbol{p}_{\boldsymbol{Dx}}$ | Per patient visit to a provider, probability of being correctly diagnosed with TB | | | 0.81 [0.75 – 0.89] | Assumed uniform prior of [0.75, 0.90] |
| $\boldsymbol{\tau}$ | Per-capita rate of first-line treatment completion | | | 2 | Corresponds to treatment duration of 6 months |
| $\boldsymbol{\delta}$ | Per-capita rate of first-line treatment interruption | | | 0.84  [0.76 – 0.93] | Assumed treatment completion follows uniform prior of [0.75 – 0.95] |
| Demographics | | | | | |
| $\boldsymbol{\mu}^{\left( \boldsymbol{j} \right)}$ | Per-capita rate of background (non-TB-related) mortality | | $h=0$ (HIV -ve) $h=2$   (on ART) | 1/64 | Mean lifespan of 64 years (World Bank) |
|  |  |  | $h=1$ (HIV +ve, not on ART) | 0.030  [0.014 – 0.053] | Calibrated to yield correct prevalence of HIV, with uniform prior range of [0 - 2] |
| $\boldsymbol{b}$ | Rate of population replenishment through birth | | | Chosen to maintain constant population by balancing all deaths | |

## 1.3 Central Asia/Europe model

In the following equations, the superscript $k$ denotes drug resistance status, with values 0, 1, denoting respectively: drug-sensitive, and rifampicin-resistant TB.

Uninfected (U):

$$\frac{dU}{dt}=b-(\lambda^{\left( 0 \right)}+\lambda^{\left( 1 \right)}+\mu)U$$

Latent, ‘fast’ progression ($L_{f}$):

$$\frac{dL_{f}^{\left( k \right)}}{dt}=\lambda^{\left( k \right)}U+c\lambda^{\left( k \right)}\sum_{k} \left( L_{s}^{\left( k \right)}+R_{lo}^{\left( k \right)}+R_{hi}^{\left( k \right)}+R^{\left( k \right)} \right)-\left( u+v+\mu\right)L_{f}^{\left( k \right)}$$

Latent, ‘slow’ progression ($L_{s}):$

$$\frac{dL_{s}^{\left( k \right)}}{dt}=vL_{k}^{\left( f \right)}-\left( w+\mu+c\lambda^{\left( 0 \right)}+c\lambda^{\left( 1 \right)} \right)L_{s}^{\left( k \right)}$$

Active TB ($I$):

$$\frac{dI^{\left( k \right)}}{dt}=uL_{f}^{\left( k \right)}+wL_{s}^{\left( k \right)}+\rho_{lo}R_{lo}^{\left( k \right)}+\rho_{hi}R_{hi}^{\left( k \right)}+\rho_{st}R_{st}^{\left( k \right)}-\left( \mu_{TB}+\sigma+\gamma\right)I^{\left( k \right)}$$

Presented for care and awaiting diagnosis with public or private sector ($D$):

$$\frac{dD^{\left( k \right)}}{dt}=\gamma I^{\left( k \right)}+\tilde{\gamma}E^{\left( k \right)}-\left( \sigma+\mu_{TB}+md \right)D^{\left( k \right)}$$

Temporarily dropped out of careseeking due to missed diagnosis ($E$):

$$\frac{dE^{\left( k \right)}}{dt}=md\left( 1-p_{Dx} \right)D^{\left( k \right)}+\tau_{1}(1-p_{SLtransf})F^{\left( 1 \right)}-\left( \mu_{TB}^{\left( k \right)}+\tilde{\gamma} \right)E^{\left( k \right)}$$

Diagnosed and initiated on first-line TB treatment ($F^{\left( j \right)}$):

*Drug-sensitive TB* ($F^{\left( 0 \right)}$):

$$\frac{dF^{\left( 0 \right)}}{dt}=mdp_{Dx}D^{\left( 0 \right)}-\left( \tau_{1}+\delta_{1} \right)T^{\left( k \right)}$$

*Rifampicin-resistant TB* ($F^{\left( 1 \right)}$):

$$\frac{dF^{\left( 1 \right)}}{dt}=mdp_{Dx}\left( 1-p_{DST} \right)D^{\left( 1 \right)}-\left( \tau_{1}+\delta_{1} \right)F^{\left( 1 \right)}$$

Diagnosed and initiated on second-line TB treatment ($S^{\left( 1 \right)}$, assumed only for rifampicin-resistant TB):

$$\frac{dS^{\left( 1 \right)}}{dt}=mdp_{Dx}p_{DST}D^{\left( 1 \right)}+\tau_{1}p_{SLtransf}F^{\left( 1 \right)}-\left( \tau_{2}+\delta_{2} \right)S^{\left( 1 \right)}$$

Recovered after treatment completion, with low relapse risk ($R_{lo}$):

$$\frac{dR_{lo}^{\left( k \right)}}{dt}=\tau_{1}T^{\left( k \right)}-\left( \mu+\rho_{lo}+c\lambda^{\left( 0 \right)}+c\lambda^{\left( 1 \right)}+h \right)R_{lo}^{\left( k \right)}$$

Recovered after treatment non-completion with high relapse risk ($R_{hi}$):

$$\frac{dR_{hi}^{\left( k \right)}}{dt}=\delta_{1}T^{\left( k \right)}-\left( \mu+\rho_{hi}+c\lambda^{\left( 0 \right)}+c\lambda^{\left( 1 \right)}+h \right)R_{hi}^{\left( k \right)}$$

Long-term recovered, following stabilisation of relapse risk ($R_{st}$):

$$\frac{dR_{st}^{\left( k \right)}}{dt}=h\left[ R_{lo}^{\left( k \right)}+R_{hi}^{\left( k \right)} \right]-\left( \mu+\rho_{st}+c\lambda^{\left( 0 \right)}+c\lambda^{\left( 1 \right)} \right)R_{st}^{\left( k \right)}$$

Force-of-infection ($\lambda^{\left( k \right)}$):

$$\lambda^{\left( 0 \right)}=\beta\left[ I^{\left( 0 \right)}+D^{\left( 0 \right)}+E^{\left( 0 \right)} \right]$$

$$\lambda^{\left( 1 \right)}=q_{RR}\beta\left[ I^{\left( 1 \right)}+D^{\left( 1 \right)}+E^{\left( 1 \right)}+F^{\left( 1 \right)} \right]$$

**Table C. Table of parameters for the Central Asia/Europe model.** When drawn from the literature, prior distributions for parameter values are assumed to follow a uniform distribution with a standard assumed range of +/-25% around the central value reported in the literature. Where no prior source exists (e.g. the probability of successful diagnosis by a private provider), uniform priors are chosen to cover an assumed, plausible range.

| Symbol | Meaning | | Value | Source |
| --- | --- | --- | --- | --- |
| Natural history | | | | |
| $\boldsymbol{\beta}^{\left( \boldsymbol{k} \right)}$ | Transmission rate (annual number of infections per case) | $k=0$ (drug sensitive) | 25.9  [21.2 – 29.8] | Model calibration |
|  |  | $k=1$ (rifampicin-resistant) | 21.3  [16.3 – 25.4] |  |
| $\boldsymbol{u}$ | Per-capita rate of ‘fast’ progression to active TB | | 0.073  [0.064 – 0.102] | Menzies et al (2018) ([4]), assuming a uniform prior distribution over +/- 25% of reported values |
| $\boldsymbol{v}$ | Per-capita rate of stabilisation from ‘fast’ to ‘slow’ latent states | | 0.84  [0.67 – 1.08] |  |
| $\boldsymbol{w}$ | Per-capita rate of ‘fast’ progression to active TB | | 0.00065  [0.00049 – 0.000474] |  |
| $\boldsymbol{\mu}_{\boldsymbol{TB}}$ | Per-capita rate of death from untreated TB | | 0.40  [0.28 – 0.58] | Tiemersma et al (2011) [5], with assumed uniform priors over a range of +/-25% |
| $\boldsymbol{\sigma}$ | Per-capita rate of self-cure from untreated TB | | 0.17  [0.13 – 0.20] |  |
| $\boldsymbol{\rho}_{\boldsymbol{lo}}$ | Per-capita rate of relapse in two years following treatment completion | | 0.033  [0.025 – 0.040] | Romanowski (2019)[6], Menzies (2009) [7] and Weis (1994) [8], with uniform prior using intervals of ± 25% |
| $\boldsymbol{\rho}_{\boldsymbol{hi}}$ | Per-capita rate of relapse in two years following treatment non-completion, or self-cure | | 0.14  [0.11 – 0.17] |  |
| $\boldsymbol{\rho}_{\boldsymbol{st}}$ | Per-capita rate of relapse beyond two years post treatment/self-cure | | 0.0015  [0.0013 – 0.0018] | Most relapse occurs in first two years after recovery: Guerra-Assuncao (2015) [9] |
| $\boldsymbol{h}$ | Per-capita annual rate of ‘stabilising’ from high to low relapse risk | | 0.5 |  |
| $\boldsymbol{c}$ | Reduced risk of reinfection arising from prior exposure | | 0.77  [0.57 – 0.90] | Andrews et al (2012) [10], with assumed uniform prior over range of [0.5 – 0.9] |
| Health services | | | | |
| $\boldsymbol{\gamma}$ | Per-capita rate of first careseeking, active TB | | 5.90  [3.92 – 15.1] | Model calibration, assuming uniform prior range of [0.1 – 100] |
| $\tilde{\boldsymbol{\gamma}}$ | Per-capita rate of repeat careseeking, active TB | | 10.4  [1.24 – 20.4] | Model calibration, assuming uniform prior range of [1 – 24] |
| $\boldsymbol{m}$ |  | |  |  |
| $\boldsymbol{d}$ | Per-capita rate of provider offering diagnosis | | 52 | Assumption, equivalent to 1 week |
| $\boldsymbol{p}_{\boldsymbol{Dx}}$ | Per patient visit to a provider, probability of being correctly diagnosed with TB | | 0.81  [0.75 – 0.89] | Assumed uniform prior of [0.75, 0.95] |
| $\boldsymbol{\tau}$ | Per-capita rate of first-line treatment completion | | 2 | Corresponds to treatment duration of 6 months |
| $\boldsymbol{\delta}$ | Per-capita rate of first-line treatment interruption | | 0.80  [0.76 – 0.92] | Assumed treatment completion follows uniform prior of [0.75 – 0.95] |
| Demographics | | | | |
| $\boldsymbol{\mu}$ | Per-capita rate of background (non-TB-related) mortality | | 1/75 | Mean lifespan of 75 years (World Bank) |
| $\boldsymbol{b}$ | Rate of population replenishment through birth | | Chosen to maintain constant population by balancing all deaths | |

# 2. Model calibration

*Calibration to pre-pandemic data*

For each country group, calibration targets are listed in Table 2 in the main text. We fitted parameters for a log-normal probability distribution to each of the targets shown. For a given country group, we then defined the overall likelihood function as a product of all relevant probability distributions. In practice, working in log-likelihood space, we computed the logarithm of the probability density, and summed over all relevant outputs (e.g. incidence, mortality and notifications, for the South Asia country group). For prior distributions on model parameters, we adopted uniform distributions as listed in Tables A – C.

For a given parameter set $\theta$, we first simulated the model to equilibrium, in the absence of HIV, drug resistance, and public sector services (assuming that all pre-DOTS TB services were comparable to the private sector today).

- For the South Asia country group, we then simulated the expansion of public sector services from 2000 to 2010.

- For the sub-Saharan Africa country group, we modelled the emergence of HIV from the point of the first available data, adjusting the rate $r_{HIV}$ in a time-dependent way in order to capture estimates (derived from the Thembisa model) for annual HIV incidence. We also modelled ART, approximating its increase to current levels of uptake in a linear way.

- For the Central Asia/Europe country group, we modelled the emergence of rifampicin resistance in 1980.

Simulating each model to 2019, we evaluated the log-posterior density as described above.

To sample from the posterior density, we used adaptive Bayesian Markov Chain Monte Carlo (MCMC) [11], an approach that uses the covariance matrix of already-sampled parameters to inform the proposal distribution. We performed this MCMC for 25,000 iterations. After discarding the burn-in and drawing every 50^th^ sample, we finally obtained 250 samples from the posterior distribution. In all model projections, we estimated 95% Bayesian credible intervals by calculating 2.5^th^ and 97.5^th^ percentiles, and median estimates using the 50^th^ percentiles.

*Capturing COVID-related disruptions*

We adopted the same approach as currently being used by WHO in their estimates of global TB burden [12]: that is, using quarterly notification data from Q1 2020 onwards, and assuming that any reductions in notifications – relative to 2019 – are attributable to reductions in diagnosis and treatment initiation. We obtained notification data at the country group level by aggregating over all countries within each group, weighted by country population. We then adjusted the term $m\left( t \right)$ in the model equations on a quarterly basis, in such a way that the timeseries of median, modelled quarterly treatment initiations would match the data for quarterly notifications as closely as possible (see Fig B).

# 3. Intervention descriptions

*Private sector engagement* (South Asia group only): Private providers do not always report TB to public health authorities. Moreover, some private providers do not follow WHO-recommended International and/or National guidelines for the diagnosis and treatment of TB, potentially leading to delays in diagnosis and lower treatment completion rates than in the public sector. Initiatives in India and elsewhere are showing how effective engagement with private providers can improve their reporting of TB, as well as encouraging uptake of molecular diagnostics and adherence support mechanisms for their patients [13,14]. Accordingly, at baseline we modelled the probability of diagnosis, per visit by a TB patient to a healthcare provider, as being lower in the private than the public sector. We also modelled patients on TB treatment in the private sector as having lower rates of completion than the NTP. As an intervention, we assumed that ‘private sector engagement’ encourages private providers to use molecular diagnostic tools, as well as offering improved adherence support to their patients (consistent with current efforts in India and elsewhere). Through these mechanisms we assumed that the accuracy of diagnosis and treatment completion rates would both improve, to the same levels as those in the public sector.

*Improved diagnostics* (all country groups): We modelled the improvement of facility-based diagnosis, which could arise from different measures including: the use of molecular diagnostic tools, potentially supported by radiographic screening and AI technologies, to identify those eligible for testing [15]; and optimising the diagnostic network, aligning the placement of diagnostic tools with areas of greatest need, as well as instating sample referral systems to further improve access to these diagnostics [16]. We assumed that the net effect of all these interventions would be to increase the probability of successful diagnosis, per visit by a symptomatic patient to a healthcare provider to 90%, amongst all public and engaged private providers. We did not consider the potential for false-positive diagnoses in our algorithm (which is relevant for costing).

*Upstream case-finding* (all country groups): Here, by ‘upstream’ we denote all stages of infectious TB disease prior to a patient’s first contact with the healthcare system, for their TB symptoms. Measures to identify TB during this period may include active (community-based) case-finding activities; intensified case-finding efforts amongst defined risk groups; or even demand generation for TB services encouraging those with symptoms to come forward for care [17]. We modelled this intervention as a 30% reduction in the delay before a patient’s first careseeking attempt. To simplify the model, we assumed that those initiating TB treatment through this route would have the same treatment outcomes as individuals receiving services without benefit of improved diagnostics or case-finding.

*Improve coverage of upfront drug sensitivity testing* (CEE group only): Not all TB patients receive a test for rifampicin resistance. Consequently, individuals with rifampicin-resistant TB may be initiated on inappropriate and ineffective first-line treatment, with the risk of being infectious and transmitting the disease to others as long as the individual is on this treatment. As an intervention, we assumed an increased use of molecular diagnostic tools, or phenotypic drug sensitivity testing, and subsequent increase in the proportion of rifampicin-resistant TB diagnoses that are initiated on appropriate, second-line treatment. We modelled their effect as an increase in the proportion of patients initiated on appropriate (first- or second-line) therapy, to 90%.

*Preventive therapy* (all country groups): We modelled the scale-up of preventive therapy amongst all people living with HIV, and all close contacts of TB patients. Consistent with the 3HP regimen [18], we assumed preventive therapy would produce a 60% relative reduction in the risk of developing TB amongst those with TB infection.

*Mass vaccination* (all country groups): Ultimately, there will be a need for prevention on a population level, not solely amongst risk groups. We assumed that a post-exposure vaccine (i.e. protecting those already infected with TB from progressing to active disease) would be licensed and introduced from 2025 onwards. We assumed the vaccine would have 60% efficacy in reducing incidence, and that vaccine-induced immunity would last on average for 10 years [19]. We denoted ‘coverage’ as the proportion of individuals with TB infection that have vaccine-induced immunity. A limitation of the model is that it does not capture different age groups separately: we were thus unable to model targeted vaccination amongst adults and adolescents alone, such as in ref [20]. For each country group, we modelled what vaccination coverage would be sufficient – in combination with all of the interventions described above – to meet the 2030 milestones.

# 4. Supplementary figures and tables

**Fig A. Results of model calibrations for all country groups.** Points in red show calibration data from 2019, drawn from the WHO Global TB Report, and aggregated over all countries in each country group. Points in blue show model projections, together with uncertainty 95% Bayesian credible intervals.


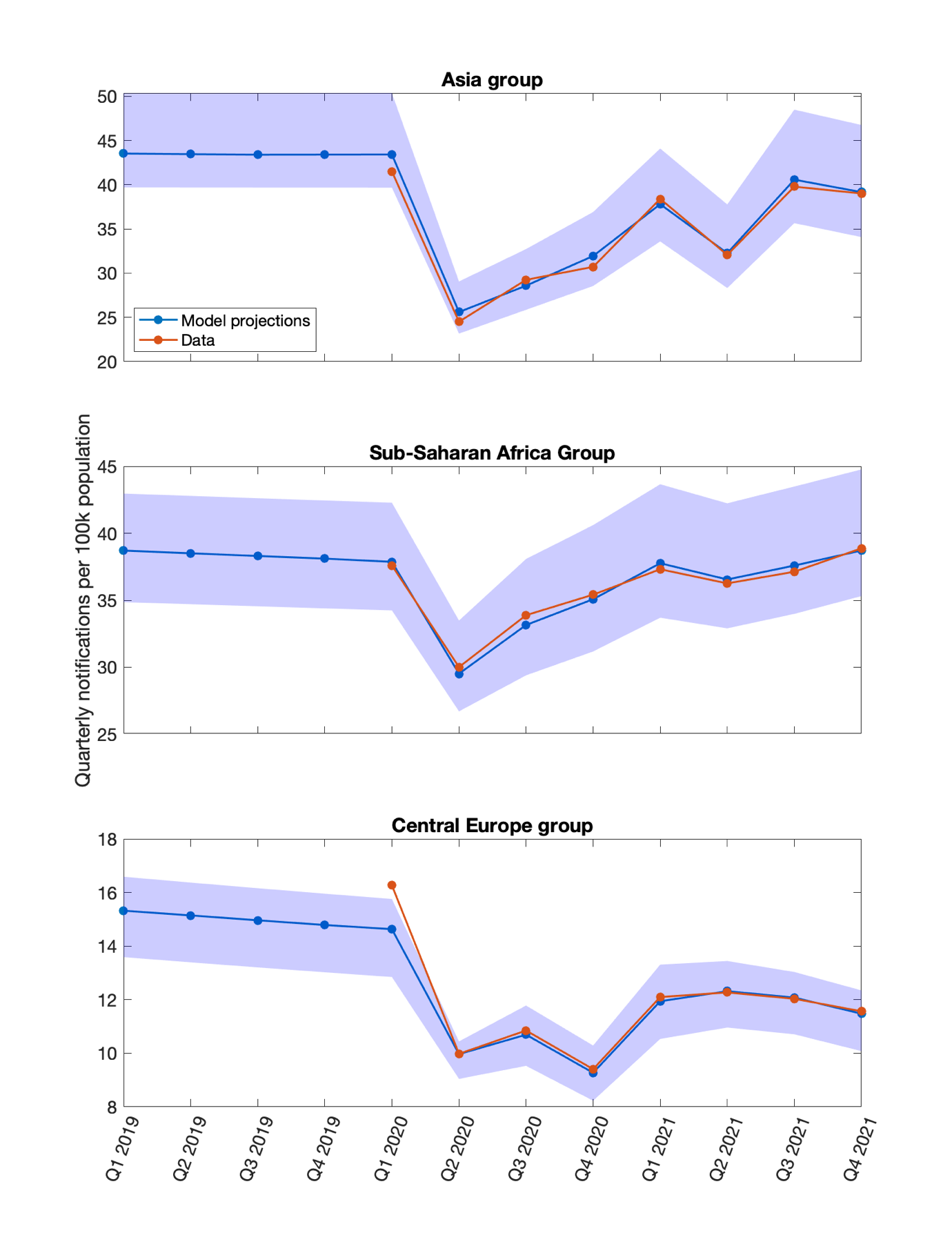
 **Fig B. COVID-related reductions in TB notifications** in all country groups, when aggregated over all countries in this group and expressed as quarterly rates per 100,000 population. Figure illustrates agreement between model projections (in blue) and data (in red). Shaded areas show 95% uncertainty intervals in model projections.


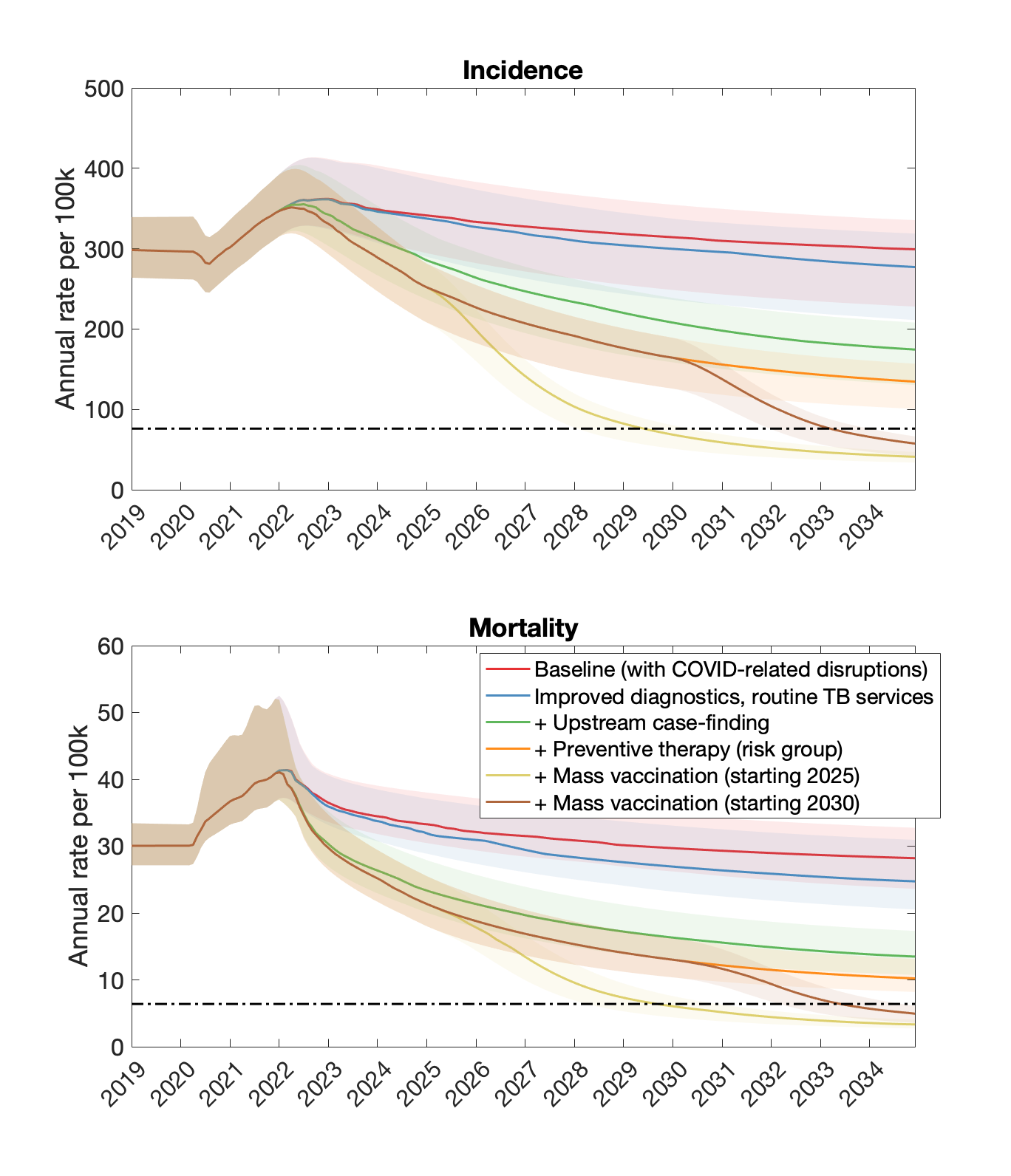


**Fig C. Incidence and mortality implications of different scenarios for vaccine development (South Asia country group)**. In each panel, the bottom two curves (in yellow and brown) show scenarios where the vaccine is deployed in 2025, as in the main text, and in 2030, with the same coverage assumptions as in Fig 2 in the main text (i.e. sufficient coverage that 72% of people with TB infection have vaccine-induced immunity). Results illustrate how delayed vaccine deployment would also lead to delayed achievement of the SDG goals: in the scenario shown here (brown curve), the goals are only achieved in 2033.

# 5. Implications of the 2018 UN HLM targets for case detection

Notably*,* disruptions due to COVID-19 present challenges in reaching the HLM case detection targets. In the South Asian country group, Fig D shows an unanticipated side effect of these disruptions: that is, that the case detection targets seem as if they will be met between 2022 – 2026 *without* any of the interventions listed above. The reason for this is twofold:

- First, this country group was already close to reaching the case detection target before COVID-19 ( Fig D, left panel, blue solid line vs dashed line).
- Second, model projections suggest that COVID-19 related disruptions in this country group have substantially increased TB incidence. Consequently, TB caseload would be inflated to such an extent that this country group would meet the case detection targets, without need for any further interventions (Fid D, left panel, red line).


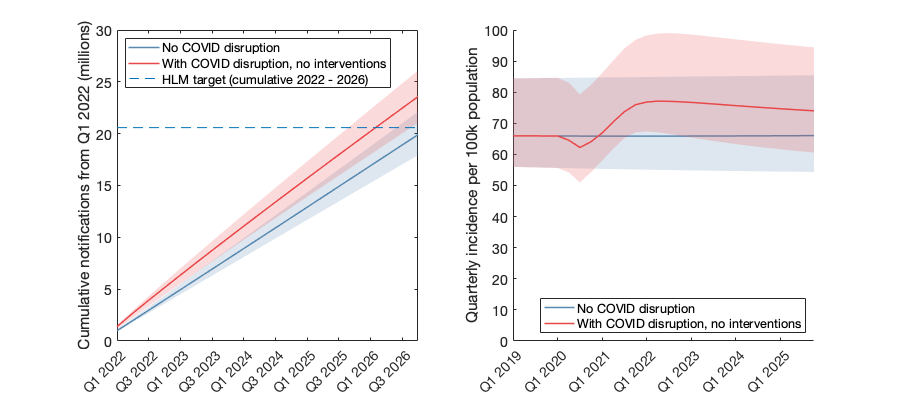
Clearly, there is no public health benefit in reaching the targets in this way.

**Fig D. Illustration of dynamics in absence of interventions in South Asia country group.** (A) Cumulative notifications from 2022, comparing scenarios of no COVID disruption (blue) with COVID disruption, but no interventions (red). At existing levels of case detection, the South Asia country group is already close to meeting the HLM targets for the cumulative number of TB patients treated by the end of 2026 (dashed line). However, by increasing TB incidence and therefore inflating TB caseload, COVID disruptions mean that the targets would be met without interventions (red curve). (B) Dynamics of incidence over time, illustrating how TB burden has been increased as a result of COVID disruptions.

Similarly, Fig E illustrates that the sub-Saharan Africa country group also shows challenges in meeting the HLM notification targets. The figure shows the cumulative notifications from 2022 – 2026 that would occur when the ‘improved diagnostics’ intervention is combined with ‘upstream case-finding’, under a range of scenarios for the coverage of the latter (measured as the percent reduction that is achieved by this intervention, in the average delay to diagnosis and treatment initiation). To interpret this figure, it is helpful to note that, in general, accelerated case-finding can exert two opposing effects on notifications: on the one hand, it can *increase* notifications because of its accompanying increase in TB case detection. However, it can also *reduce* notifications because of its effect on transmission (i.e. as a result of declining incidence). Fig E suggests that, in the Sub-Saharan Africa country group, the combined effect of these two factors is that case detection would remain below the HLM targets, despite the aggressive case-finding efforts depicted at the right-hand side of the figure. Notably, these case-finding efforts have strong impact on incidence that are not reflected in the notifications. Similarly, Fig F shows how cumulative notifications between 2022 and 2026 would vary in the CAR/EU country group, under a sequence of interventions described in further detail below. Although these interventions ultimately lead to achievement of the SDG goals, for reasons similar to those shown in Fig F, notifications never meet the target set for this country group (shown by the horizontal dashed line).

**Fig E. How notifications vary with case-finding efforts in the Sub-Saharan Africa country group.** For this figure, we assumed that the ‘improved diagnostics’ intervention is implemented as described in the main text, in combination with ‘upstream case-finding’. The horizontal axis shows different scenarios for the coverage of the ‘upstream case-finding’ intervention, measured as the reduction that it achieves, in the average duration that a TB patient remains culture-positive, before diagnosis and treatment initiation. The vertical axis shows cumulative TB notifications between 2022 – 2026. Overall, the figure illustrates that – in the context of this model at least – increased case-finding efforts do not necessarily lead to increases in case notification.

**Fig F. How notifications vary with interventions in the CAR/EU region**. Intervention scenarios are as described in the following section, and are shown here in successive combination. As described below, these interventions have the effect of meeting the SDG targets by 2030 in the CAR/EU country group. The horizontal dashed line shows the HLM target, assumed for the period 2022 – 2026.

Overall, these results suggest the following: in the wake of COVID disruptions, meeting the 2018 UN HLM targets appears no longer sufficient for achieving the 2030 milestones. For example in South Asia, an elevated TB burden (as a result of missed opportunities for diagnosis during the COVID response) means that HLM targets for notification might be reachable without further intervention. In Sub-Saharan Africa and CAR/EU countries, interventions may reduce incidence too rapidly, for notifications to increase to the levels envisaged in the HLM targets.

# 6. References

1 Dye C, Glaziou P, Floyd K, *et al.* Prospects for tuberculosis elimination. *Annu Rev Public Health* 2013;**34**:271–86. doi:10.1146/annurev-publhealth-031912-114431

2 Abu-Raddad LJ, Sabatelli L, Achterberg JT, *et al.* Epidemiological benefits of more-effective tuberculosis vaccines, drugs, and diagnostics. *Proc Natl Acad Sci* 2009;**106**:13980–5. doi:10.1073/pnas.0901720106

3 Stop TB Partnership. Global Plan to End TB, 2023 - 2030. 2022. https://www.stoptb.org/global-plan-to-end-tb/global-plan-to-end-tb-2023-2030

4 Menzies NA, Wolf E, Connors D, *et al.* Progression from latent infection to active disease in dynamic tuberculosis transmission models: a systematic review of the validity of modelling assumptions. Lancet Infect. Dis. 2018. doi:10.1016/S1473-3099(18)30134-8

5 Tiemersma EW, van der Werf MJ, Borgdorff MW, *et al.* Natural History of Tuberculosis: Duration and Fatality of Untreated Pulmonary Tuberculosis in HIV Negative Patients: A Systematic Review. *PLoS One* 2011;**6**:e17601. doi:10.1371/journal.pone.0017601

6 Romanowski K, Balshaw RF, Benedetti A, *et al.* Predicting tuberculosis relapse in patients treated with the standard 6-month regimen: an individual patient data meta-analysis. *Thorax* 2019;**74**:291–7. doi:10.1136/thoraxjnl-2017-211120

7 Menzies D, Benedetti A, Paydar A, *et al.* Effect of duration and intermittency of rifampin on tuberculosis treatment outcomes: A systematic review and meta-analysis. PLoS Med. 2009;**6**. doi:10.1371/journal.pmed.1000146

8 Weis SE, Slocum PC, Blais FX, *et al.* The Effect of Directly Observed Therapy on the Rates of Drug Resistance and Relapse in Tuberculosis. *N Engl J Med* 1994;**330**:1179–84. doi:10.1056/NEJM199404283301702

9 Guerra-Assunção JA, Houben RMGJ, Crampin AC, *et al.* Recurrence due to relapse or reinfection with Mycobacterium tuberculosis: a whole-genome sequencing approach in a large, population-based cohort with a high HIV infection prevalence and active follow-up. *J Infect Dis* 2015;**211**:1154–63. doi:10.1093/infdis/jiu574

10 Andrews JR, Noubary F, Walensky RP, *et al.* Risk of progression to active tuberculosis following reinfection with Mycobacterium tuberculosis. *Clin Infect Dis* 2012;**54**:784–91. doi:10.1093/cid/cir951

11 Haario H, Saksman E, Tamminen J. An Adaptive Metropolis Algorithm. *Bernoulli* Published Online First: 2007. doi:10.2307/3318737

12 World Health Organization. Global Tuberculosis Report 2021. https://www.who.int/teams/global-tuberculosis-programme/tb-reports

13 Arinaminpathy N, Deo S, Singh S, *et al.* Modelling the impact of effective private provider engagement on tuberculosis control in urban India. *Sci Rep* 2019;**9**:3810. doi:10.1038/s41598-019-39799-7

14 Pai M, Dewan P. Testing and treating the missing millions with tuberculosis. *PLoS Med* 2015;**12**:e1001805. doi:10.1371/journal.pmed.1001805

15 Qin ZZ, Ahmed S, Sarker MS, *et al.* Tuberculosis detection from chest x-rays for triaging in a high tuberculosis-burden setting: an evaluation of five artificial intelligence algorithms. Lancet. Digit. Heal. 2021;**3**:e543–54. doi:10.1016/S2589-7500(21)00116-3

16 Albert H, Purcell R, Wang YY, *et al.* Designing an optimized diagnostic network to improve access to TB diagnosis and treatment in Lesotho. *PLoS One* 2020;**15**:e0233620. doi:10.1371/journal.pone.0233620

17 Burke RM, Nliwasa M, Feasey HRA, *et al.* Community-based active case-finding interventions for tuberculosis: a systematic review. *Lancet Public Heal* 2021;**6**:e283–99. doi:10.1016/S2468-2667(21)00033-5

18 Sterling TR, Villarino ME, Borisov AS, *et al.* Three Months of Rifapentine and Isoniazid for Latent Tuberculosis Infection. *N Engl J Med* 2011;**365**:2155–66. doi:10.1056/NEJMoa1104875

19 Tait DR, Hatherill M, Der Meeren O Van, *et al.* Final analysis of a trial of M72/AS01E vaccine to prevent tuberculosis. *N Engl J Med* Published Online First: 2019. doi:10.1056/NEJMoa1909953

20 Knight GM, Griffiths UK, Sumner T, *et al.* Impact and cost-effectiveness of new tuberculosis vaccines in low- and middle-income countries. *Proc Natl Acad Sci* 2014;**111**:15520–5. doi:10.1073/pnas.1404386111
