# Supplementary material for: Meeting the 2030 END TB goals in the wake of COVID-19: A modelling study of countries in the USAID TB portfolio
Source: PLOS Glob Public Health. 2023 Oct 23;3(10):e0001271. doi: 10.1371/journal.pgph.0001271 (PMC10593207; doi:10.1371/journal.pgph.0001271)
Supplement: Attachment — Submitted filename: Reviewer responses.docx [file pgph.0001271.s002.docx]

**Responses to reviewer and Editor comments**

We thank the reviewers for their careful reading and constructive comments on our manuscript. Below, we provide point-by-point responses to each comment, with revisions shown as ‘tracked’ changes in the manuscript.

**Editor comments**

*The study is very important for global public health. Please consider the suggestions and address the concerns given by both reviewers. Furthermore, Pre-COVID calibration data was used in this study. I am wondering, should you consider the modelling fitting step for the data as 'model training' or 'model calibration'?*

Thank you for the comment – our preference would be to refer to this as ‘model calibration’, as the term ‘training’ nowadays is arguably better reserved for statistical models such as machine learning. We also note that while pre-COVID data was used for calibration, this is only the first of two steps: the second step uses data during the COVID pandemic to capture the disruptions that occurred during this time. We have added to the Methods to emphasise the continuity between these two steps. Please see line 104, “In the second step, we used the posterior distribution sampled in the first step, to simulate the model to the end of 2019…”. We hope the Editor will agree that this text helps to clarify that the calibration was not limited to pre-COVID data.

**Reviewer 1** [Summary comments omitted]

*1. Page 2, introduction, the authors should provide a short discussion about the general picture of TB modelings.*

We have added to the text to give a short overview of modelling: in order to maintain the overall flow, we have limited the scope of this overview to modelling that is relevant to the SDGs and End TB goals. Please see line 36, “Mathematical modelling has been a helpful tool…”, and the text that follows.

*2. Page 3, when the authors discussed the models, they should provide part of the models in the main text and refer to the full models in the supporting information.*

We agree with the reviewer that the Methods should show an overview, with technical details provided in the supporting information: however, we note that the current methods section is already structured along these lines. It provides an overview of the model that can be understood without mathematical equations, which themselves are provided in the supporting information. We hope the reviewer will agree that this structure is accessible to the general audience for this journal, while also including sufficient technical detail to be reproducible.

*3. In the supporting information, the explanation of the models is not complete. It does not show the uniqueness of the models they applied.*

Thank you – we have now added to the supporting information to highlight that the main innovation of this modelling is to combine interventions with post-COVID modelling. Please see supporting information, first page, “While several modelling studies have addressed intervention priorities…” The revised introduction also now makes this point more clearly, by setting our work in the context of previous, relevant modelling work.

*4. Page 3, “For simplicity we did not aim to model each country individually, and instead modelled interventions at the level of the country groups shown in table 1.” The authors consider three country groups so did the three groups interact with others? Also, within each country group, are the populations in different countries mixed together to be considered in the model? It seems that it does not satisfy the real situation?*

This is indeed a relevant point: the role of cross-border movement in spreading infection is an under-developed question in modelling, and addressing this question fully is outside the scope of our current work. Nonetheless, we have added to the text to clarify this important point as a limitation. Please see line 265, “One important limitation in our analysis…”.

*5. Do the ranges of the values in Table 2 come from the real data or depend on some assumption?*

We have added to the caption for Table 2 to clarify that the ranges come from WHO published estimates. Please see line 301, “All data, including uncertainty intervals,…”

*6. Are there any reference to decide the ranges of the model parameters? For example, some of them were set +/- 10%, and some were +/-25%.*

Our apologies for the typo – the table should actually show +/-25% on all parameters, and has now been corrected as such. Please see the updated table S3, supporting information. We have also added to the caption to explain the standard assumptions underlying these ranges. Please see caption, “When drawn from the literature, …” and the text that follows.

**Reviewer 2** [Summary comments omitted]

*Disclosures: The submitting author states ‘This work was supported by USAID. YDM, AB, CV and SA are employees of USAID; the funders otherwise had no role in the study’. Given they are all authors on the manuscript, and I am sure they contribute more than enough to take on that role, this disclosure must be untrue. Suggest modifying to state the role in funding and authorship more clearly.*

We note that the author contributions were specified separately during the submission process, as follows: NA: Formal analysis, Investigation, Methodology, Writing – original draft and review & editing; YDM, AB, CV: Conceptualization, Validation, Writing – review and editing; SA: Conceptualization, Investigation, Validation, Writing – original draft and review & editing. Thus it may not be necessary to re-state this information in the disclosure.

Nonetheless, to avoid confusion, we have amended the disclosure as follows (with edit shown underlined): “This work was supported by USAID. YDM, AB, CV and SA are employees of USAID, and their respective contributions are specified in the author information. The funders otherwise had no role in the study.”

*Line 113 ‘We assumed all interventions would be rolled out starting in 2022’*

*Not true. Vx later. Needs correcting*

Thank you – we have now corrected this. Please see line 127, “…with the exception of vaccination, which was assumed to be rolled out starting in 2025.”

*Line 118. Unlike the rest of the manuscript, this is a rather wordy and unclear sentence. Could it be made more clear?*

We have amended the sentence, and hope the reviewer will agree that it is now much clearer. Please see line 132, “The 2018 targets related to cumulative case-finding…”

*Line 161. Starting “In each fig…” Delete sentence. Not needed. These #s are quoted in the subsequent text*

Now deleted. Please see line 176.

*Line 167. It would be better (IMHO) to move the explanatory text on why lower vx coverage is required in SSA from the discussion into the results. As it is based on an analysis of model results, it could fairly be defined as a result, and that is where the reader will have the question*

We note that this sentence (now in line 180) in the results is only pointing out the fact that SSA requires less vaccination coverage than the other regions. The explanation is left to the discussion (see paragraph starting line 249). Also given the relevance of this sentence to the reviewer’s previous comment, we would prefer to leave it as it is.

*Line 168 - required coverage if 2030 vx roll out assumed. It would be useful to add the coverages assumed (100%?) and year the 2030 targets met, into the main text, for all regions. This is an important point given the long delays to the start of the M72 Ph3 trial*

We have added to the text to clarify that this figure assumes the same coverage levels as in Figure 2, i.e. sufficient vaccination coverage that ultimately, 72% of people with TB infection have vaccine-induced immunity. Please see line 183, “…but with the same levels of coverage and pace of rollout.”

Please also see the supporting information, caption to Fig.S3, “…with the same coverage assumptions as in Figure 2 in the main text…”

*Line 176 - notifications no longer reliable. It needs a couple more sentences here in the main text to say why notifications no longer reliable (not just in supporting material)*

We have added to the text to mention briefly why notifications may be no longer reliable. Please see line 192, “In particular, in the South Asia country group, increased notifications…”

*Line 238 - would be sensible to add something like ‘or more widespread use of safer preventive therapy’, to this sentence*

We have added to the text to mention this. Please see line 258, “…as well as widened use of shortened, simplified and safe preventive therapy.”

*Line 255. Would be great to add something like ‘, and mobilsing the $5bn/ year that is also needed for R&D to get the new vaccine etc, else targets certainly won't be met’*

An important point. We have added to the text accordingly. Please see line 282, “Importantly, the Global Plan also calls for an investment of at least USD 5bn…”

*Line 274. Table. For row ‘TB preventive therapy, risk groups’, need to add the assumed efficacy to the description box.*

Now added. Please see Table 3, p.10, “…assuming 60% efficacy…”

*Line 274. Table. For row ‘TB vaccine’, need to add the assumed duration of efficacy, & that coverage was calculated to meet End TB targets, to the description box*

Now added. Please see bottom row of Table 3, p.10.
